# Epigenetic silencing of miR-338 facilitates glioblastoma progression by de-repressing the pyruvate kinase M2-β-catenin axis

**DOI:** 10.18632/aging.101271

**Published:** 2017-08-02

**Authors:** Bo Han, Xiangqi Meng, Hui Chen, Lingchao Chen, Xing Liu, Hongjun Wang, Daming Liu, Fei Gao, Lin Lin, Jianguang Ming, Bo Sun, Shi Yin, Ruijia Wang, Pengfei Wu, Jinquan Cai, Chuanlu Jiang

**Affiliations:** ^1^ Department of Neurosurgery, The Second Affiliated Hospital of Harbin Medical University, Harbin 150086, China; ^2^ Neuroscience Institute, Heilongjiang Academy of Medical Sciences, Harbin 150086, China; ^3^ Chinese Glioma Cooperative Group (CGCG), Beijing 100050, China; ^4^ Department of Laboratory Diagnosis, The Second Affiliated Hospital of Harbin Medical University, Harbin 150086, China; ^5^ Beijing Neurosurgical Institute, Beijing 100050, China; ^6^ Department of Neurosurgery, Huashan Hospital, Fudan University, Shanghai 200040, China

**Keywords:** glioblastoma, epigenetic modification, MiR-338, PKM2, β-catenin

## Abstract

Glioblastoma is the most malignant type of brain tumor, and its high invasiveness and multiplication severely shortens patients’ overall survival. The embryonic pyruvate kinase M2 (PKM2) isoform is highly expressed in human cancer. We used computational target gene prediction*, in vitro* cell culture, immunoblotting, quantitative real-time PCR, ATP measurements, luciferase reporter assays, wound-healing assays, Transwell assays, RNA immunoprecipitation PCR, co-immunoprecipitation, flow cytometry and tumor xenografts to study the regulation of the PKM2/β-catenin axis in glioma. *PKM2* was predicted to be a potential target of miR-338. MiR-338 was downregulated in high-grade gliomas due to hypermethylation of CpG islands in its promoter, and ectopic expression of miR-338 inhibited cell proliferation, invasion and ATP generation. MiR-338 inhibited PKM2 expression by binding to the 3′ untranslated region of *PKM2*, which ultimately prevented binding of PKM2 to β-catenin and reduced T-cell factor/lymphoid enhancer factor reporter gene transcriptional activity. MiR-338 also inhibited PKM2 expression, attenuated glioma growth and prolonged survival in an animal model. These results confirm that miR-338, a tumor suppressor, suppresses the PKM2/β-catenin axis and is downregulated in glioblastoma. This provides a theoretical basis for glioma-targeting therapy.

## INTRODUCTION

Malignant primary brain tumors are major causes of cancer-related death in children and adults. Glioblastoma, the most common and lethal type of primary intracranial tumor, is highly infiltrative, progresses rapidly, and is relatively resistant to both radiotherapy and common chemotherapeutic agents.

Even glioblastoma patients receiving aggressive comprehensive treatments have a median survival of less than 14 months. Though the study of the clinical and pathological subtypes of glioblastoma has improved in recent years, the mechanism of glioma progression remains unclear [1].

Epigenetic phenotypes are those that cannot be explain-explained by changes in the DNA sequence. Epigenetics often refers to changes in a chromosome that alter gene activity and expression, including DNA methylation and histone modification. CpG-island methylation is one of the epigenetic alternations that regulates gene expression. Hypermethylation of CpG islands often causes gene silencing [2].

MicroRNAs (miRNAs) are small non-coding RNAs with lengths of approximately 22 nucleotides. Most mature miRNAs couple with the RNA-induced silencing complex (RISC) and bind the 3′ untranslated regions (3′-UTRs) of their target mRNAs [3-5]. This binding downregulates gene expression by either inhibiting translation or promoting mRNA degradation. Maladjusted miRNAs may function as oncogenes or tumor suppressors. In addition, miRNAs have important functions in various biological processes, including development, differentiation and metabolism.

The embryonic pyruvate kinase M2 (PKM2) isoform is widely expressed in cancer cells [6]. While the involvement of PKM2 in aerobic glycolysis and the Warburg effect has been established [7-9], the non-metabolic functions of PKM2 are still being revealed [7, 10-12]. In this study, we analyzed several public datasets to determine the correlation of *PKM2* expression with glioma grades. We also examined the effects of ectopic miR-338 expression on glioma cell growth, invasiveness and metabolic activity. Finally, we investigated the effects of miR-338 on epidermal growth factor (EGF)-induced cell cycling, PKM2/β-catenin binding and β-catenin transcriptional activity *in vitro*, and on the survival of mice bearing tumors *in vivo*.

## RESULTS

### *PKM2* expression correlated with glioma grade progression and promoted glioma cell proliferation and invasion

**Figure 1:**
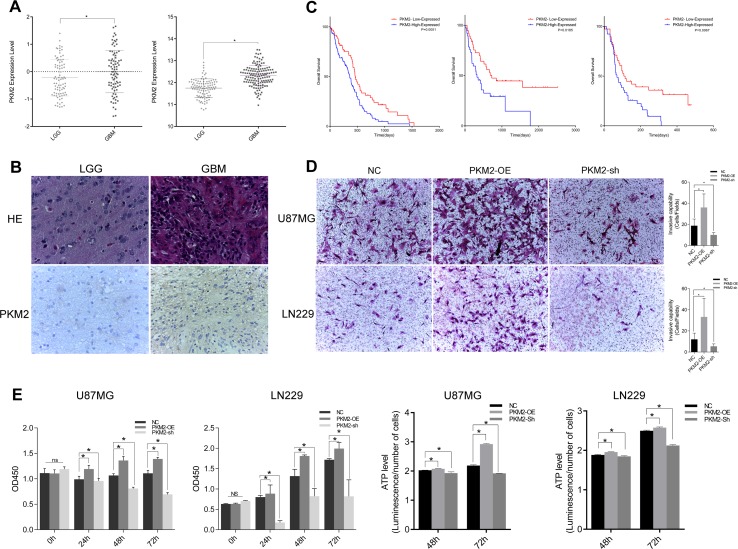
PKM2 correlated with glioma grade progression and promoted glioma cell proliferation and invasion (**A**) *PKM2* expression and its correlation with glioma grade prognosis in the CGGA and REMBRANDT. (**B**) IHC staining depicting the difference in PKM2 expression between low‐grade and high‐grade gliomas. (**C**) Kaplan‐Meier survival curves indicating cumulative survival as a function of time for patients with high versus low PKM2 expression. (**D**) Representative images of Transwell assays of cells after transfection. Number of invading cells shown as a histogram (P<0.05). (**E**) Cell viability was examined with a CCK‐8 assay at different time intervals after transfection. (**F**) ATP level assay at different time intervals after transfection (P<0.05).

After analyzing discovery sets from public databases, we found that the expression of *PKM2* increased with the WHO glioma grade (Figure 1A, p=0.0002). An IHC assay further confirmed that PKM2 expression was elevated in high-grade gliomas (Figure 1B). We then evaluated the prognostic value of PKM2, and found that low *PKM2* expression was favorably associated with survival in glioma patients (Figure 1C, P<0.05); these results were supported byGSE4271, GSE4412.

We also carried out a Transwell chamber assay to explore the effect of PKM2 on invasiveness. Over-expression of PKM2 enhanced the invasiveness of glioma cells, while inhibition of PKM2 reduced it (Figure 1D). A Cell Counting Kit-8 (CCK-8) assay also demonstrated that PKM2 overexpression promoted glioma cell proliferation, whereas PKM2 inhibition slowed cell growth (Figure 1E). Cells overexpressing PKM2 generated larger amounts of ATP, while cells transfected with sh-*PKM2* generated lower amounts of ATP (Figure 1F).

### MiR-338 binds to and degrades *PKM2* transcripts through the RISC

**Figure 2:**
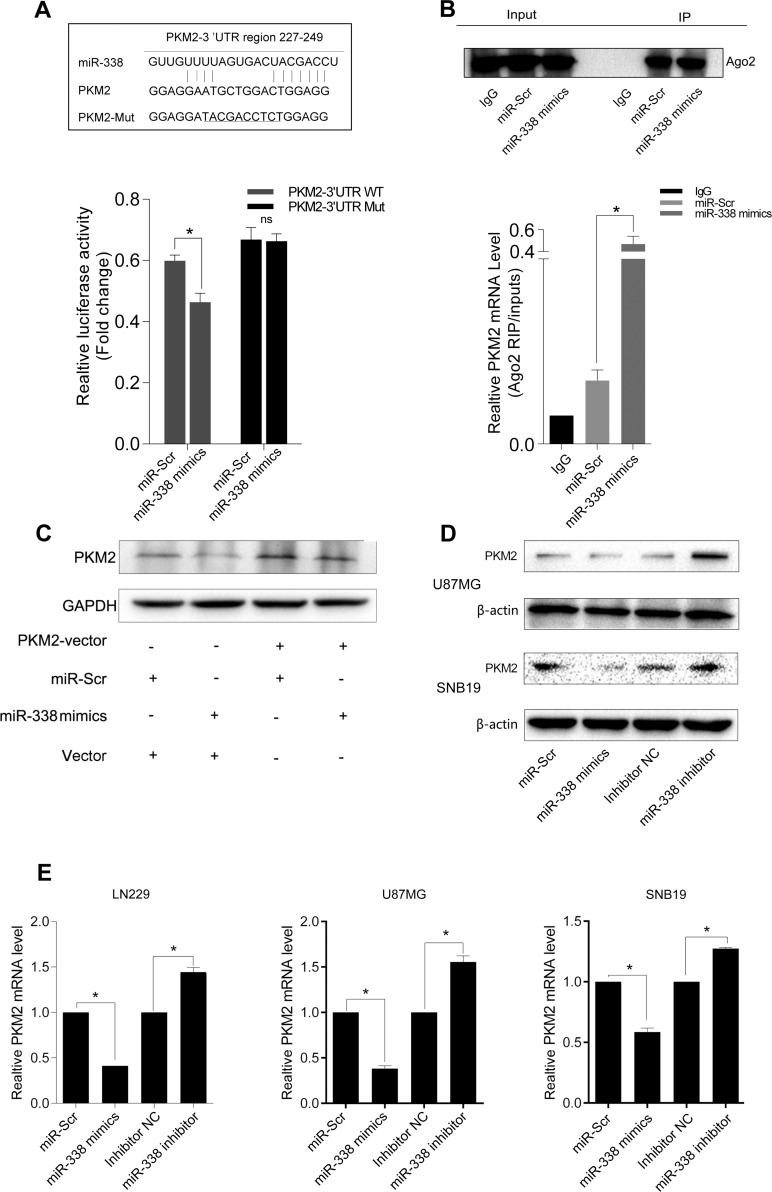
MiR‐338 binds to and degrades *PKM2* transcripts through the RISC (**A**) Graphic of the seed sequence of miR‐338 matched with the 3′‐UTR of the *PKM2* gene, and the design of wild‐type or mutant *PKM2* 3′‐UTRs containing reporter constructs. Luciferase reporter assays in glioma cells after co‐transfection of cells with wild‐type or mutant *PKM2* 3′‐UTRs and miRNA. The data represent the fold‐change in the expression (mean and standard error) of three replicates (P<0.05). (**B**) Western blot of AGO2 protein immunoprecipitated from cell extracts with an AGO2 antibody, or IgG. The amount of *PKM2* bound to AGO2 or IgG was measured by qrt‐PCR in the presence of miR‐338 mimics or miR‐Scr (P<0.05). (**C**) Western blot of the effect of miR‐338 overexpression on PKM2 protein expression after cells were transfected with the *PKM2* plasmid or plain vector. (**D**) Western blot of PKM2 expression 48 hours after cells were transfected with miR‐Scr/miR‐ 338 or with inhibitor‐NC/miR‐338 inhibitor. (**E**) Qrt‐PCR of *PKM2* mRNA expression 48 hours after transfection (P<0.01).

Then, we sought the miRNAs that potentially downregulate *PKM2* expression through direct binding to sites in the 3′-UTR of the *PKM2* transcript. We identified the potential binding sites between miR-338 and *PKM2* in miRbase and TargetScan. Then, we inserted the potential binding sites into a pmirGLO dual luciferase reporter vector (Figure 2A). As shown in Figure 2A, luciferase reporter activity was significantly lower in LN229 cells co-transfected with pmirGLO-*PKM2*-WT and the miR-338 mimic than in those co-transfected with pmirGLO-*PKM2*-WT and the negative control (Figure 2A, P<0.05). Luciferase reporter activity did not change when cells were co-transfected with the miR-338 mimic and pmirGLO-*PKM2*-MUT (Figure 2A).

We next used RIP assays to further test the association between *PKM2* and miR-338, and found that *PKM2* transcripts could be detected in AGO2 complexes. *PKM2* transcript levels in AGO2 complexes increased significantly when cells were treated with miR-338 mimics, indicating that miR-338 might regulate *PKM2* expression through the RISC (Figure 2B, P<0.01).

To explore whether miR-338 downregulates PKM2 protein expression in glioma cells, we co-transfected cells with the *PKM2* overexpression plasmid and miR-338 mimics, as shown in Figure 2C. Overexpression of miR-338 inhibited PKM2 expression, but over-expression of PKM2 restored PKM2 protein levels (Figure 2D & E). These results revealed that miR-338 inhibits PKM2 expression by directly binding to its mRNA.

### MiR-338 expression inversely correlated with glioma malignancy and was restrained by CpG-island methylation

**Figure 3:**
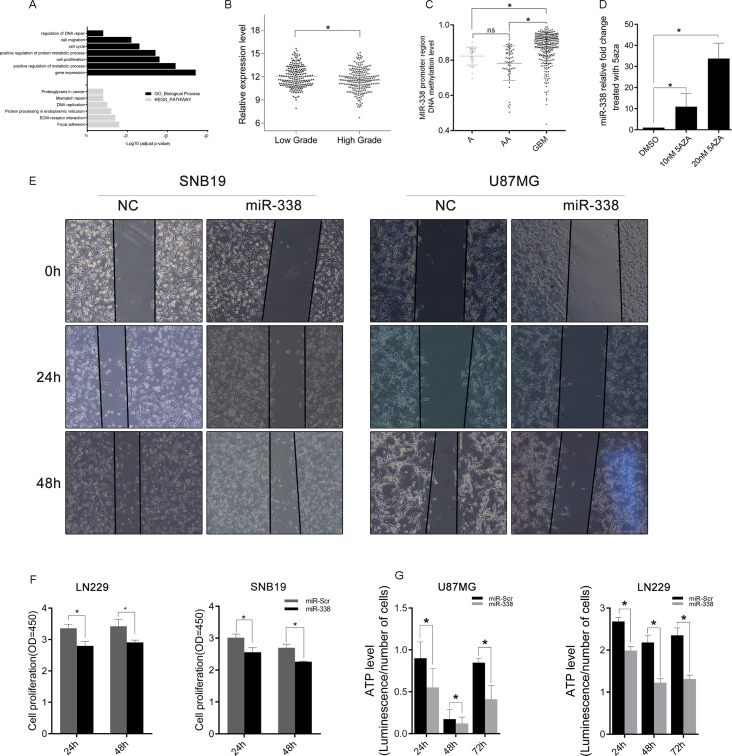
MiR‐338 expression inversely correlated with glioma malignancy and was restrained by CpG‐island methylation (**A**) Gene Ontology function analysis and KEGG pathway enrichment of miR‐338‐downregulated genes. (**B**) The expression difference of miR‐338 between low‐grade and high‐grade gliomas in TCGA. (**C**) The methylation level of the miR‐338 promoter region between low‐grade and high‐grade gliomas in TCGA. (**D**) Qrt‐PCR of miR‐338 expression 48 hours after cells were treated with dimethyl sulfoxide or 5‐azacytidine (P<0.01 to 10nM 5aza, P<0.05 to 10nM 5aza). (**E**) Wound‐healing assay; the scratch was photographed at 0 h, 24 h and 48 h after transfection. (**F**) Cell viability was examined with a CCK‐8 assay at different time intervals after transfection (P<0.05, P<0.05, P<0.05, P<0.01, respectively). (**G**) Cellular ATP levels in SNB19 and LN229 glioma cell lines normalized with cell numbers 24 h, 48 h and 72 h post‐transfection with miR‐Scr or miR‐338 (P<0.01).

We then analyzed the function of miR-338-related genes in TCGA glioblastoma datasets, and discovered that miR-338-downregulated genes were enriched in metabolic processes, the cell cycle and cell migration processes (Figure 3A). KEGG pathway analysis revealed that miR-338 was negatively related with the extracellular matrix-receptor interaction pathway and the DNA replication pathway, indicating that miR-338 may function as a tumor suppressor in glioblastoma. In TCGA datasets, miR-338 expression was lower in high-grade gliomas than in low-grade gliomas (Figure 3B), and methylation of the miR-338 promoter was higher in glioblastoma than in low-grade glioma (Figure 3C).

Next, we treated SNB19 cells with the DNA methyl-transferase inhibitor 5-azacytidine, and found that miR-338 was upregulated (Figure 3D, P<0.01 to 10nM 5aza, P<0.05 to 10nM 5aza). We also examined the effects of miR-338 on the proliferation and migration of glioma cells. CCK-8 and wound-healing assays revealed that miR-338 could significantly inhibit cell proliferation and migration (Figure 3E & F). In addition, ATP levels were lower in miR-338-treated glioma cells than in miR-Scr-treated cells (Figure 3G).

### MiR-338 reduced the binding between PKM2 and β-catenin to repress β-catenin transcriptional activity

**Figure 4:**
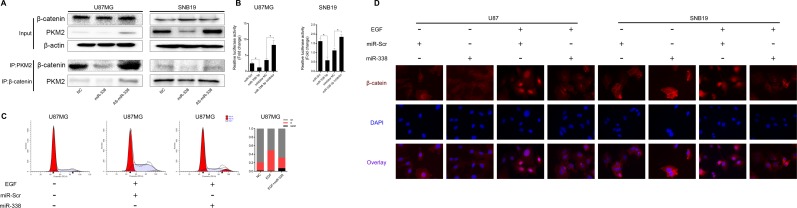
MiR‐338 reduced the binding between PKM2 and β‐catenin to repress β‐catenin transcriptional activity (**A**) Co‐IP assay to investigate the effect of miR‐338 on the binding between PKM2 and β‐catenin. (**B**) U87 and SNB19 cells were transiently transfected (24 h) with the pGL4.74 plasmid and co‐transfected with pGL4.75. Cells were then treated as indicated for 48 h. Both firefly and Renilla luciferase activities were calculated and recorded as fold‐induction (P<0.05, P<0.05). (**C**) Cell cycle analysis of miR‐Scr/miR‐338‐transfected cells treated with EGF, and overview of the cell cycle. (**D**) Immunostaining of β‐catenin location after miR‐Scr/miR‐338‐ transfected cells were treated with EGF.

PKM2, a key protein in the Warburg effect [13], has been reported to affect β**-**catenin transcriptional activity and participate in tumorigenesis [14-16]. In addition, PKM2 can regulate β-catenin transcriptional activity upon EGF stimulation [11]. We tested the association between PKM2 and β-catenin through co-IP assays. The binding between PKM2 and β-catenin was weakened when miR-338 was overexpressed, but was enhanced when miR-338 expression was inhibited (Figure 4A).

Moreover, the transcriptional activity of β-catenin was lower in miR-338-transfected cells than in control cells (Figure 4B). Twenty-four hours after U87 cells were transfected with miR-338 and co-treated with EGF, cell cycle analysis demonstrated that miR-338 jeopardized EGF-induced cell cycling (Figure 4C). Immuno-fluorescence also revealed that miR-338 prevented EGF-dependent β-catenin translocation to the nucleus (Figure 4D). These results suggested that miR-338 may attenuate the binding between PKM2 and β-catenin to reduce T-cell factor/lymphoid enhancer factor transcriptional activity after EGF stimulation, thus inhibiting cell proliferation.

### MiR-338 inhibited tumor growth *in vivo* and prolonged the survival of mice bearing tumors

**Figure 5:**
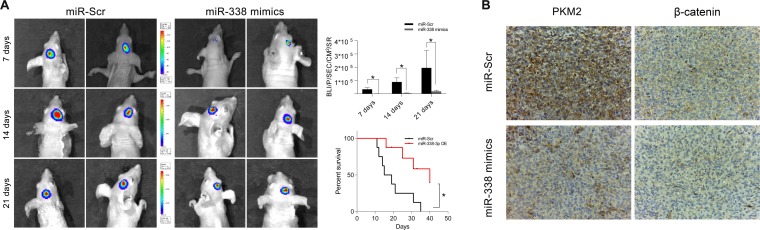
MiR‐338 inhibited tumor growth *in vivo* and prolonged the survival period (**A**) Luminescence imaging for miR‐338‐ treated U87‐luc tumors versus miR‐Scr‐treated controls (P<0.01, P<0.01, P<0.05, respectively). Kaplan‐Meier survival curves indicating that outcomes were significantly better in mice transfected with miR‐338 than in mice transfected with miR‐Scr (P<0.01). (**B**) IHC staining revealed that PKM2 and β‐catenin protein levels were lower in the miR‐338‐treated group than in the miR‐Scr‐treated group.

We then built a tumor-bearing animal model by intracranially transplanting U87-luc cells into mice, and used a bioluminescence imaging system to determine the tumor volumes. Tumor volumes were considerably lower in mice treated with miR-338-overexpressing U87 cells than in mice treated with miR-Scr-expressing U87 cells. Survival analysis revealed that survival was also better in the miR-338 overexpression group than in the control group (Figure 5A). IHC staining of tumor sections confirmed that miR-338 overexpression could inhibit PKM2 protein expression *in vivo*. Interestingly, β-catenin protein levels were also reduced upon miR-338 overexpression (Figure 5B).

## DISCUSSION

Although there have already been several studies on miRNAs and their cancer-related functions, little has been known about the involvement of miRNAs in the PKM2-β-catenin axis. PKM2 is widely expressed in human cancers and has critical functions in several types of human neoplasia, including prostate cancer, promyelocytic leukemia and glioma [17-19]. PKM2 is also a key kinase in the Warburg effect, the main energy reaction in cancer cells; thus, disrupting PKM2 expres-sion could suppress tumorigenesis. Earlier studies indicated that PKM2 could be upregulated by mTOR and that disruption of PKM2 expression could suppress mTOR-induced tumorigenesis [20]. However, in recent years, PKM2 has been found to exert a great influence on cancer cells, including on non-metabolic pathways, cell cycling, transcription factor distribution and chemotherapy resistance [21-23]. PKM2 can also regulate the Wnt signaling pathway by combining with β-catenin and adjusting its transcriptional activity, and can regulate gene transcription as a protein kinase that phosphorylates histone H3 [8, 12]. In this study, we found that PKM2 expression increased with glioma grade, and that glioblastoma patients with higher PKM2 expression had worse outcomes. Exogenous expression of PKM2 increased glioma cell proliferation, invasiveness and metabolism, while inhibition of PKM2 expression restrained these effects.

MiRNAs are small, endogenous, noncoding RNAs that can alter the biological behavior of cells by regulating target gene expression. MiRNAs downregulate gene expression by binding to the 3′-UTRs of mRNAs, leading to their degradation. MiR-338 has been reported as a tumor suppressor in various kinds of tumors [24–26]. Several genes have been identified as the targets of miR-338, including *NRP1*, *RAB14* and *HIF-1α* [25, 27, 28]. In our study, using bioinformatic prediction tools, functional experiments *in vitro* and luciferase reporter assays, we determined that miR-338 could bind to *PKM2* transcripts. As previous studies have demonstrated that mature miRNAs couple with the RISC to bind to the 3′-UTRs of their target mRNAs, we performed a RIP-PCR assay, which confirmed that miR-338 could degrade *PKM2* mRNA through the RISC.

Genome methylation is an important regulator of gene expression. Hypermethylation of gene promoter CpG islands is one cause of gene downregulation [29]. In this study, we found that miR-338 expression was lower in glioblastoma than in low-grade glioma, corresponding to the higher level of miR-338 promoter region methylation in glioblastoma. MiR-338 expression was upregulated when cells were treated with the DNA methyltransferase inhibitor 5-azacytidine. Over-expression of miR-338 significantly reduced the migratory and proliferative capabilities of glioma cells.

Our results also demonstrated that PKM2 could bind to β-catenin and induce the Wnt signaling pathway upon EGF stimulation, while overexpression of miR-338 reduced the activity of the Wnt signaling pathway *in vitro* in a luciferase reporter assay. In glioma cells treated with EGF, overexpression of miR-338 reduced the binding between β-catenin and PKM2. One of the downstream functions of the Wnt signaling pathway is the induction of cell cycling [30, 31]. A lower percentage of EGF-treated glioma cells were observed to be in S phase after transfection with miR-338 than after transfection with miR-Scr. Ultimately, the transfection of glioma cells with miR-338 mimics in an orthotopic model reduced the cellular tumorigenicity and increased the survival of nude mice, which might have been due to the inhibition of PKM2 expression.

In summary, we found that the miR-338, as a suppressive miRNA in gliomas, inhibited proliferation, cell cycling and ATP generation by downregulating PKM2, thus preventing the binding between PKM2 and β-catenin. This research provides theoretical evidence for glioma-targeting therapy.

## MATERIALS AND METHODS

### Ethics statement

Informed consent was obtained from all patients involved in this study, and the study protocol was approved by the Clinical Research Ethics Committee of The Second Affiliated Hospital of Harbin Medical University. The protocol for animal studies was also approved by the Clinical Research Ethics Committee of The Second Affiliated Hospital of Harbin Medical University. In the present study, all the datasets were from the following public websites: The Chinese Glioma Genome Atlas (CGGA, http://www.cgga.org.cn/) [32], GSE4271, GSE4412 (https://www.ncbi.nlm.nih.gov), The Cancer Genome Atlas (TCGA, https://cancergenome.nih.gov/) and The Repository for Molecular Brain Neoplasia Data (REMBRANDT, http://cabig.cancer.gov/solutions/conductresearch/rembrandt).

### Cell lines and culture conditions

Human glioma cell lines (U87, LN229, SNB19) were purchased from the Chinese Academy of Sciences Cell Bank. All cells were cultured in Dulbecco's modified Eagle's medium (DMEM)/F12 (Corning) supplemented with 10% fetal bovine serum (Bioind) and 1% antibiotic (Sigma) at 37°C in a humidified atmosphere with 5% CO_2_ and 95% air. Cells were passaged every two days.

### Plasmids and miRNA pairs

Pre-miR-338, Anti-sense of miR-338(define as AS-miR-338) plasmids and plain vectors were purchased from Vigen Company (Shandong, China). The *PKM2* plasmid, short hairpin (sh)-*PKM2* plasmid and plain vector were kindly provided by Lingchao Chen, M.D. (Department of Neurosurgery, Huashan Hospital, Fudan University). The miR-338 inhibitor, mimic and corresponding control RNA were obtained from Ribobio Company (Guangzhou, China; see Supplementary data for detailed sequences). The pmirGLO-*PKM2*-wild-type (WT) and–mutant (MUT) reporter plasmids were purchased from GenePharma (Shanghai, China). The pGL4.49 [luc2P/TCF-LEF RE/Hygro] and pGL4.74 [hRluc/TK] vector plasmids were purchased from Promega. Lipofectamine 2000 (Invitrogen) was used to transfect cells with the plasmids, scrambled miRNA (miR-Scr), miR-338, inhibitor-negative control (NC), or miR-338 inhibitor according to the manufacturer's instructions.

### Wound-healing assay and Transwell invasion assay

A vascular mimicry assay was performed as described previously [33]. In brief, cells were seeded in six-well plates and cultured until confluent. Then, the cells were transfected with plasmids, and the cell monolayer was scraped with a 200-microliter sterile pipette tip to create a scratch. The cells were washed twice with phosphate-buffered saline and then incubated in DMEM/F12 without FBS. Photographs of the scratched area were taken at 0 h, 6 h and 24 h through a Leica DM750 microscope, and were analyzed with Image-pro software. The scratches were captured in six different photographs.

The Transwell invasion assay was performed in 24-well cell culture chambers with Transwell inserts (Corning) with 8-μm pores that were pre-coated with Matrigel. In brief, cells transfected with *PKM2*/sh-*PKM2*/Vector-plasmid were seeded at a density of 5 × 10^4^ cells per upper well in 200 μL of culture medium (DMEM/F12, 4% FBS), and the lower chamber was filled with 500 μL of medium (DMEM/F12, 50% FBS). After 24 hours, the upper surface was removed by scrubbing with a cotton-tipped stick, while the lower surface was fixed with methanol for five minutes, air-dried and stained with hematoxylin and eosin. All experiments were performed in triplicate.

### Quantitative real-time PCR (qrt-PCR)

Total RNA was extracted with RNAiso Plus Reagent (TaKaRa), and cDNA was synthesized with a PrimeScript RT Reagent Kit (TaKaRa) according to the manufacturer's instructions. Then, qrt-PCR was performed in triplicate with a CFX-96 Real-Time System (Bio-Rad). Endogenous mRNA levels of *PKM2* were determined with a SYBR PrimeScript RT-PCR Kit (Roche) and normalized to those of *β-actin* as an endogenous control. Quantification of miR-338 was performed with a stem-loop real-time PCR miRNA kit (Ribobio). The qrt-PCR data were analyzed by the 2^−△△Ct^ method. (See Supplementary material for the qrt-PCR primers.)

### Western blotting, immunofluorescence, immunoprecipitation and immunohistochemistry assays

Western blotting, immunofluorescence and immuno-histochemistry (IHC) assays were performed as previously described [34-36]. Rabbit anti-PKM2 (1:1000, Cell Signaling Technology), rabbit anti-β-catenin (1:1000, Cell Signaling Technology), anti-β-actin and anti-GAPDH antibodies (1:1000, Zsbio) and horseradish peroxidase-labeled secondary antibodies (1:4000, Zsbio) were used for Western blotting. Co-immunoprecipitation (co-IP) assays were performed with PureProteome Protein A/G Mix Magnetic Beads (Merck Millipore) as described in the manufacturer's protocol, and the samples were analyzed through Western blotting. For the co-IP assays, cells were transfected with the Pre-miR-Vector, Pre-miR-338 or AS-miR-338 for 48 h, and then were extracted and collected with radioimmunoprecipitation assay buffer. Rabbit antibodies against PKM2, β-catenin (1:200, Cell Signaling Technology) and Alexa Fluor® 594-labeled secondary antibody (1:1000, Invitrogen) were used for IF and IHC.

### Flow cytometry assays

Cells were transfected with the Pre-miR-Vector or Pre-miR-338 for 48 h, and then were treated with EGF for 6 h (100 ng/mL, Novus). The medium was replaced with serum-free medium for 24 h, and the cells were collected and fixed with 75% ethanol at 4°c overnight. The supernatant was discarded and the cells were washed twice with ice-cold phosphate-buffered saline. The cells were re-suspended in 500 μL propidium iodide (BD) staining buffer for 30 min at room temperature. Stained cells were analyzed on a FACSCanto II (BD).

### ATP measurement

A total of 3000 exponential-phase cells were plated into each well of a 96-well plate (100 μL medium/well) and cultured overnight. Then, the cells were transfected with miR-338 mimics or control mimics, inhibitor RNA or control RNA for another 48 h. The assays were conducted with a Kinase-Glo® Luminescent Kinase Assay (Promega) according to the manufacturer's protocol. Data were expressed as fold-changes.

### Tumor xenograft study

In brief, miR-338-overexpressing cells and control cells (3 × 10^5^ cells per mouse in 3 μL total) transfected with a luciferase lentivirus were injected intracranially into five-week-old female nude mice, as described earlier (n=8/group). After eight days, tumors were measured by bioluminescence with an IVIS Lumina Imaging System (Xenogen). Cryosections (4 mm) were stained and used for IHC. These procedures were performed following approval by the Harbin Medical University Institutional Animal Care and Use Committee.

### RNA-binding protein immunoprecipitation (RIP)

RIP experiments were performed with a Magna RIP RNA-binding Protein Immunoprecipitation Kit (Millipore, Catalogue Number 17-700) and an AGO2 antibody (Abcam, Catalogue Number ab57113) according to the manufacturer's instructions. Co-precipitated RNAs were subjected to qrt-PCR analysis.

### Luciferase reporter assay

Cells were seeded at 2×10^3^ cells/well in 96-well plates and allowed to settle overnight. The next day, cells were co-transfected with pmirGLO-*PKM2*-WT or -MUT reporter plasmids and a miR-338 mimic or inhibitor. Twenty-four hours after transfection, cell lysates were prepared, and luciferase reporter activity was quantified with a Dual-Luciferase Reporter Assay System (Promega).

For the measurement of TCF/LEF activity, cells were seeded at 2×10^3^ cells/well in 96-well plates and allowed to settle overnight. The next day, cells were transfected with miR-Scr, miR-338- mimics, inhibitor-NC, miR-338 inhibitor. Forty-eight hours after transfection, cells were co-transfected with pGL4.49 [luc2P/TCF-LEF RE/Hygro] and pGL4.74 [hRluc/TK] vector plasmids. Cell lysates were prepared twenty-four hours after second transfection and luciferase reporter activity was quantified with a Dual-Luciferase Reporter Assay System (Promega).

Data were expressed as fold-changes from the control.

### Statistical analysis

According to the cutoff value (the most significant split) [37], patients in our dataset were stratified into the PKM2-high group and the PKM2-low group. Student's t test and the Chi-square test were used to determine the significance of differences between the two groups. Overall survival curves were plotted according to the Kaplan–Meier method, and the log-rank test was applied for comparison. All differences were considered statistically significant at the level of P<0.05. Statistics were performed with SPSS Graduate Pack 19.0 statistical software (SPSS, Chicago, IL, USA) or GraphPad Prime 15.0.

## SUPPLEMENTARY MATERIAL TABLES


