# Supplementary material for: Epigenetic silencing of miR-338 facilitates glioblastoma progression by de-repressing the pyruvate kinase M2-β-catenin axis
Source: Aging (Albany NY). 2017 Aug 2;9(8):1885–97. doi: 10.18632/aging.101271 (PMC5611983; doi:10.18632/aging.101271)

SUPPLEMENTARY MATERIAL

Supplementary Table S1. The sequence of real-time PCR primers.

Gene	Forward Sequence	Reverse Sequence
β-actin	CTGGGACGACATGGAGAAAA	AAGGAAGGCTGGAAGAGTGC
PKM2	GAACATCCTGTGGCTGGACT	GCACCTTTCTGCTTCACCTG

Supplementary Table S2. The sequence of miRNA mimics，miRNA Inhibitor，miR-Scr，Inhibitor NC.

plain vector	UUUGUACUACACAAAAGUACUG
hsa-miR-338	UCCAGCAUCAGUGAUUUUGUUG
AS-miR-338	CAACAAAAUCACUGAUGCUGGA
hsa-miR-338-3p mimic	positive-sense strand 5’UCCAGCAUCAGUGAUUUUGUUG 3’<br>Antisense strand 3’AGGUCGUAGUCACUAAAACAAC 5’
hsa-miR-338-3p inhibitor	5’mCmAmAmCmAmAmAmAmUmCmAmCmUmGmAmUmGmCmUmGmGmA 3’
miR-Scr	positive-sense strand 5’-UUUGUACUACACAAAAGUACUG-3’<br>Antisense strand 3’-AAACAUGAUGUGUUUUCAUGAC-5'
Inhibitor NC	5’- mCmAmGmUmAmCmUmUmUmUmGmUmGmUmAmGmUmAmCmAmAmA-3

Supplementary Table S3. pMIR plasmid profiles.

pMIR plasmid profiles

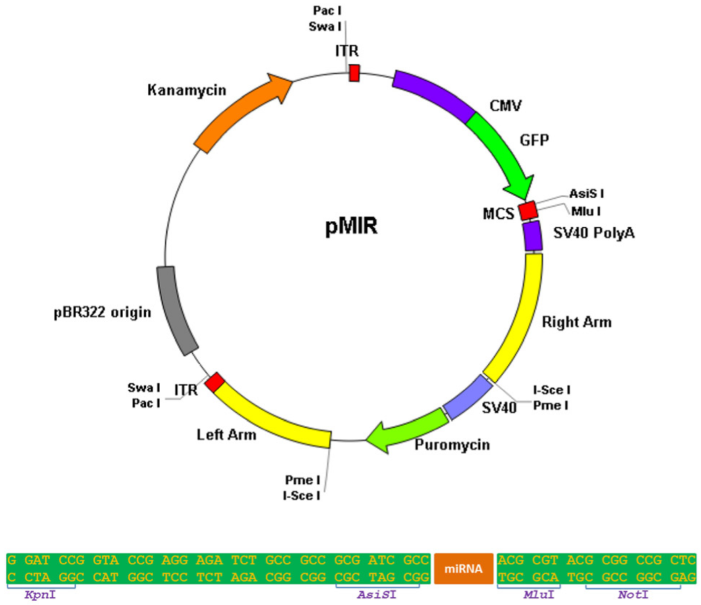

pmirGLO plasmid profiles

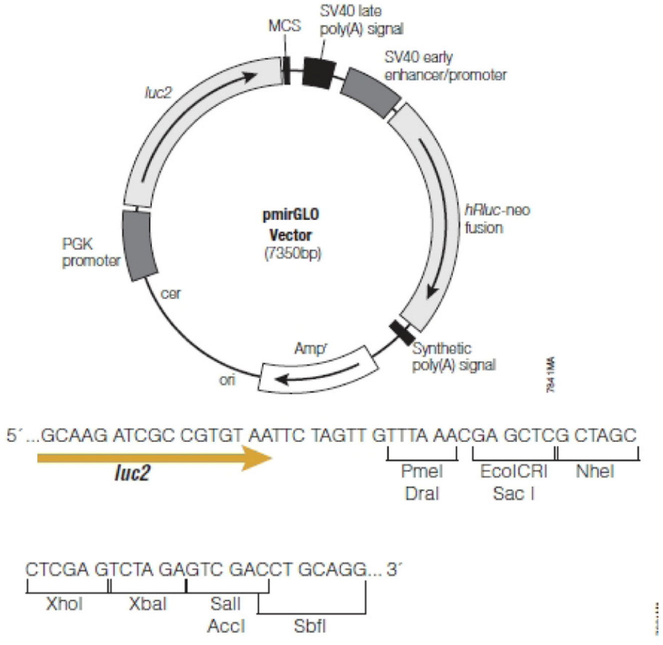

Supplement: Supplementary file 1 [file aging-09-1885-s001.pdf]
